# Maternal Serum Heme-Oxygenase-1 (HO-1) Concentrations in Early Pregnancy and Subsequent Risk of Gestational Diabetes Mellitus

**DOI:** 10.1371/journal.pone.0048060

**Published:** 2012-11-06

**Authors:** Chunfang Qiu, Karin Hevner, Daniel A. Enquobahrie, Michelle A. Williams

**Affiliations:** 1 Center for Perinatal Studies, Swedish Medical Center, Seattle, Washington, United States of America; 2 Department of Epidemiology, University of Washington School of Public Health, Seattle, Washington, United States of America; 3 Department of Epidemiology, Harvard School of Public Health, Boston, Massachusetts, United States of America; Medical Faculty, Otto-von-Guericke University Magdeburg, Germany

## Abstract

**Background:**

Heme oxygenase-1 (HO-1) concentrations have been recently reported to be elevated in impaired glucose tolerance and type 2 diabetes mellitus (T2DM). However, no study has examined the association between HO-1 concentrations and gestational diabetes mellitus (GDM).

**Methods:**

In a case-control study, nested within a prospective cohort of pregnant women (186 GDM cases and 191 women who remained eu-glycemic through pregnancy), we assessed the association of maternal serum HO-1 concentrations, measured in samples collected at 16 weeks gestation, on average, with subsequent risk of GDM. Maternal serum HO-1 concentrations were determined using ELISA. We fitted multivariate logistic regression models to derive estimates of odds ratios (ORs) and 95% confidence intervals (CIs).

**Results:**

Median serum HO-1 concentrations in early pregnancy were lower in women who subsequently developed GDM compared with those who did not (1.60 vs. 1.80 ng/mL, p-value = 0.002). After adjusting for maternal age, race, family history of T2DM and pre-pregnancy body mass index, women with HO-1≥3.05 ng/mL (highest decile) experienced a 74% reduction of GDM risk (95% CI; 0.09–0.77) compared with women whose concentrations were<1.23 ng/mL (lowest quartile).

**Conclusion:**

Serum HO-1 concentrations were inversely associated with subsequent GDM risk. These findings underscore the role of oxidative stress in the pathogenesis of GDM. Additional studies are warranted to confirm the clinical utility of serum HO-1 in diagnosis of GDM, particularly in the early pregnancy.

## Introduction

Gestational diabetes mellitus (GDM) is one of the most common pregnancy complications affecting approximately 7% of all pregnancies and up to 14% of pregnancies in high-risk populations [1]. GDM is manifested by pregnancy –induced insulin resistance and a relative impairment in insulin secretion [2]. Women with a history of GDM have a considerably elevated risk of developing impaired glucose tolerance or type 2 diabetes mellitus (T2DM) in the years following pregnancy. For instance, the cumulative incidence of developing T2DM later in life has been reported to range from 22% to 60% [3]–[5] among women with a prior history of GDM. Although the pathogenesis of GDM remains unclear, enhanced oxidative stress in the form of lipid peroxidation [6] and DNA oxidative damage [7] in GDM have been noted in previous studies. Moreover, results from other studies have indicated that chronic systemic inflammation [8], elevated leptin [9], reductions in adiponectin [10] and endothelial dysfunction [11] are important metabolic derangements that precede the clinical diagnosis of GDM.

The enzyme heme oxygenase (HO) has been implicated in several physiological functions throughout the body, including the control of vascular tone and regulation of the inflammatory and apoptotic cascades as well as contributing to the antioxidant capacity in several organ systems [12]. These various biological functions attributed to HO are mediated via the catalytic products of heme degradation, namely carbon monoxide (CO), biliverdin-derived bilirubin, and free iron (Fe^2+^) [13]. The expression of inducible isoform HO-1 is highly responsive to a broad spectrum of chemical and physical stress agents, such as hydrogen peroxide, hypoxia, hyperoxia, pro-inflammatory cytokines and heme itself. Consequently, HO-1 is regarded as a stress protein [12]. The role of HO-1 in the pathogenesis of T2DM and its complications has been investigated by several research teams. Of note, several investigators have assessed the influence of total HO activity and blood concentrations on neovascularization [14]. For example, the up regulation of HO-1 gene expression and increased HO-1 enzyme activity are thought to play protective roles against the development of diabetic complications [14]. In animal studies, investigators have shown that elevated HO-1 gene expression in pancreatic islet cells exposed to elevated glucose concentrations [15]. Of note, other investigators have shown that chronic hyperglycemia in a rat model, where hyperglycemia was induced by partially pancreatectomy, resulted in decreased HO-1 gene expressions in islets cells with increasing duration of hyperglycemia [16]. Recently, elevated circulating plasma HO-1 concentrations have been observed in patients with impaired glucose regulation [17] and T2DM [18] in a Chinese population. However, these cross- sectional, case-control study designs do not allow investigators to clarify whether alterations in HO-1 concentrations are a cause or consequence of the impaired glucose tolerance or T2DM.

To our knowledge, no previous study has examined the extent to which, if at all, maternal serum HO-1 concentrations in early pregnancy may be related with incident GDM. On the basis of an emerging literature documenting associations of HO-1 with hyperglycemia, impaired glucose tolerance and T2DM, we used serum specimens gathered as part of a prospective cohort study of women receiving prenatal care before 20 weeks gestation to examine the association between early-pregnancy serum HO-1 concentrations and subsequent risk of GDM.

## Materials and Methods

### Study Population

This nested case-control study was based on the Omega study, a prospective cohort study of risk factors of pregnancy complications [19]. In this cohort, participants were recruited from women attending prenatal care at clinics affiliated with Swedish Medical Center in Seattle and Tacoma General Hospital in Tacoma, Washington. Women were ineligible if they initiated prenatal care after 20 weeks gestation, were younger than 18 years of age, did not speak and read English, did not plan to carry the pregnancy to term, or did not plan to deliver at either of the two research hospitals. Participants completed a questionnaire administered in English by a trained interviewer at or near enrollment. These questionnaires were used to gather information on socio-demographic, anthropometric, and behavioral characteristics and reproductive and medical histories. After delivery, maternal and infant medical records were abstracted for information on the course and outcomes of pregnancy. The study protocol was approved by Institutional Review Boards of Swedish Medical Center and Tacoma General Hospital according to the declaration of Helsinki and written informed consent was obtained from all individuals. Between 1996 and 2006, 5,063 eligible women were approached and 4,000 (approximately 79%) agreed to participate. A total of 3,886 pregnant women provided blood samples and completed interviews.

From structured questionnaires and medical records, we obtained information of covariates including maternal age, educational attainment, height, pre-pregnancy weight, reproductive and medical histories, and medical histories of first-degree family members. We also collected information on annual household income and maternal smoking before and during pregnancy. Pre-pregnancy body mass index (BMI) was calculated as pre-pregnancy weight in kilograms divided by height in meters squared. Maternal medical records were reviewed to collect detailed medical and clinical information including laboratory results such as glucose concentrations from screening test and first trimester hematocrit concentrations. We used the food frequency questionnaire (FFQ) from the Women’s Health Initiative Clinical Trial [20] to assess maternal dietary intake during the three-month period that started before conception and covered the first trimester. Participants completed FFQs at an average of 15.3 weeks gestation. Dietary intake values of nutrients, and minerals including heme iron [21] were estimated using food composition tables from the University of Minnesota Nutrition Coding Center nutrient database (Nutrition Coordinating Center, Minneapolis, MN).

Maternal non-fasting blood samples, collected in 10 mL Vacutainer tubes at 16 weeks gestation, on average, were protected from ultraviolet light, kept on wet ice and processed within 20 min of blood collection. Serum decanted into cryovials was frozen at −80°C until analysis. Serum HO-1 concentrations were determined by HO-1 ELISA kits (Enzo Life Sciences Plymouth Meeting, PA) in accordance with the manufacturer’s protocol. The intra- and inter-assay coefficients of variations of HO-1 kit had been determined to be<10%. All assays were performed without knowledge of pregnancy outcome.

In our study settings, according to the recommendations from the American Diabetes Association (ADA) [22], pregnant women were screened at 24–28 weeks gestation using a 50 g 1-hour oral glucose challenge test. Those patients who failed this screening test (glucose ≥7.8 mmol/L or 140 mg/dL) were then followed-up within 1–2 weeks with a 100 g, 3-h oral glucose tolerance test (OGTT). Women were diagnosed with GDM if two or more of the 100 gram OGTT glucose levels exceeded the following thresholds based on the ADA criteria: fasting ≥5.3 mmol/L (≥95 mg/dL); 1-hour ≥10.0 mmol/L (≥180 mg/dL); 2-hour ≥8.6 mmol/L (≥155 mg/dL); 3-hour ≥7.8 mmol/L (≥140 mg/dL) [22].

We identified and sampled all 191 women who developed GDM and randomly selected (N = 191) controls among women who did not develop GDM. There were 5 GDM cases with an inadequate volume of serum for laboratory testing, thus our final analytical study population included 186 GDM cases and 191 non-GDM controls.

### Statistical Analysis

Because the distribution of serum HO-1 concentrations was not normally distributed (Skewness/Kurtosis tests for Normality was<0.01 in either GDM cases or controls), we tested differences in median serum HO-1 concentrations between cases and controls using the Mann-Whitney U test. Spearman correlation coefficients were examined between serum HO-1 concentrations with maternal characteristics, selected FFQ variables, plasma glucose concentrations after the 50-gram oral glucose challenge screening test, and first trimester hematocrit concentrations. First, we categorized serum HO-1 concentrations according to quartiles determined by the distribution among controls. We used logistic regression models to estimate odds ratios (OR) and 95% confidence interval (95% CI). We also explored the possibility of a nonlinear relation between HO-1 and GDM odds, using generalized additive logistic regression modeling procedures (GAM) [23]. S-Plus (version 6.1, release 2, Insightful Inc. Seattle, WA) was used for these analyses. We evaluated the covariates in Table 1 as potential confounders and included in the final model those that altered unadjusted ORs by 10% or more and a prior, including maternal age, race/ethnicity, family history of diabetes and pre-pregnancy body mass index. All analyses except the GAM procedure) were performed using Stata 9.0 (Stata, College Station, TX). All reported confidence intervals were calculated at the 95% level and all reported p-values are two-tailed.

**Table 1:** Characteristics of study participants according to gestational diabetes (GDM) case-control status.

	GDM (n = 186)		Controls (n = 191)		P value
	n	%	n	%	
Maternal age (years)	33.9±4.7		33.0±4.3		0.05
Maternal age (years)					
<25	7	3.8	5	2.6	0.08
25–29	19	10.2	35	18.3	
30–34	73	39.2	79	41.4	
≥35	87	46.8	72	37.7	
Race					
White	133	71.5	165	86.4	0.004
African American	5	2.7	3	1.6	
Asian	33	17.7	14	7.3	
Other	14	7.5	9	4.7	
unknown	1	0.5	0	0.0	
Maternal education<12 years	9	4.8	5	2.6	0.25
Nulliparous	104	55.9	107	56.0	0.98
Unmarried	31	16.7	28	14.7	0.59
Family History: Diabetes	59	31.7	29	15.2	<0.001
Family History: Hypertension	107	57.5	87	45.6	0.02
No Vitamin Intake in Pregnancy	3	1.6	5	2.6	0.72
Cigarette Smoking History					
Never	142	76.3	141	73.8	0.26
Quit during pregnancy	30	16.1	41	21.5	
Current	14	7.5	9	4.7	
Inactive in Pregnancy	40	21.5	30	15.7	0.15
Pre-pregnancy BMI (kg/m^2^)	26.8±7.1		23.4±5.1		<0.001
Pre-pregnancy BMI (kg/m^2^)					
<18.5	4	2.2	10	5.2	<0.001
18.5–24.9	91	48.9	132	69.1	
25–29.9	51	27.4	39	20.4	
≥30	40	21.5	10	5.2	
Gestational Age at Delivery (weeks)	38.1±2.1		38.5±2.8		0.08
Infant Birthweight (kg)	3.40±0.63		3.41±0.65		0.97
GA at Blood Collection (weeks)	15.3±2.9		15.4±3.1		0.81
Hours from last meal to blood collection	2.76±2.36		2.72±2.24		0.84
**Serum HO-1 (ng/mL), median [IQR]**	**1.60 [1.11–2.08]**		**1.80 [1.23–2.41]**		**0.002**

1 Mean ± SD (standard deviation).

## Results

**Figure 1:**
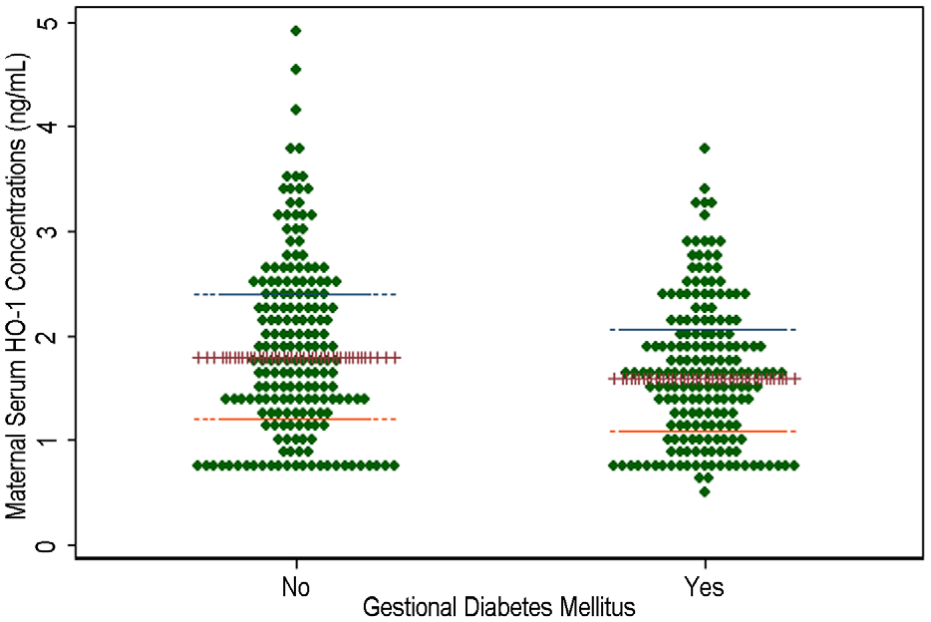
The dotplot of serum HO-1 concentrations according to GDM case-control status with median (+++), the lowest, or the highest quartile bar lines (---) indicated.

Sociodemographic, medical, and behavioral characteristics of the study population, according to case status, are presented in **Table 1**. Early pregnancy serum HO-1 concentrations were significantly lower among women who developed GDM as compared with women who did not (median 1.60 vs. 1.80 ng/mL, respectively, p-value = 0.002) (**Table 1** and **Figure 1**).

Among controls, serum HO-1 concentrations were inversely correlated with dietary heme iron intake and red/processed meat intake (Spearman ρ = –0.27 with p-value<0.001; ρ = –0.18 with p-value = 0.02, respectively) (**Table 2**). Serum HO-1 was also inversely correlated with post-load plasma glucose concentrations and first trimester hematocrit levels within the control group (Spearman ρ = –0.16 with p-value = 0.03; ρ = –0.18 with p-value = 0.02, respectively). However, similar correlations were not observed among GDM cases.

**Table 2:** The Spearman correlation coefficients (CC) between serum HO-1 concentrations and other biomarkers as well as maternal characteristics.

Spearman CC (Non-Parametric Correlations)	GDM(n = 186)		Controls(n = 191)		
	ρ	P-value		ρ	P-value
Maternal age (years)	−0.12	0.12		−0.10	0.18
Maternal pre-pregnancy BMI (kg/m^2^)	−0.11	0.15		−0.11	0.12
Maternal gestational age at blood collection (weeks)	−0.08	0.28		−0.05	0.47
Time from last meal to blood collection (hours)	0.08	0.27		0.08	0.27
Gestational age at delivery (weeks)	0.05	0.51		−0.10	0.17
Infant birth weight (kg)	0.01	0.85		−0.04	0.54
***Available valid FFQ (calories: 500–3500*** ***kcal/day)***	*N = 155*			*N = 170*	
Total energy intake (kcal/day)	−0.08	0.35		−0.05	0.53
Dietary heme iron intake (mg/day)	−0.09	0.27		−**0.27**	**<0.001**
Dietary nonheme iron intake (mg/day)	−0.09	0.29		0.01	0.85
Dietary total iron intake (mg/day)	−0.09	0.29		−0.03	0.74
Red/processed meat intake (serving/day)	0.03	0.72		−**0.18**	**0.02**
Vegetable and fruit intake (servings/day)	0.009	0.91		−0.08	0.31
Dietary fiber intake (mg/day)	−0.01	0.89		−0.05	0.53
Dietary soluble fiber intake (mg/day)	0.03	0.72		0.02	0.80
Dietary insoluble fiber intake (mg/day)	−0.03	0.74		−0.07	0.36
Egg intake (egg/weeks)	−0.14	0.10		−0.01	0.94
Cholesterol (mg/day)	−0.12	0.14		−0.13	0.09
First trimester hematocrit (%) (n = 340 available)	0.05	0.54		−**0.18**	**0.02**
Glucose concentrations after a 50 g oral glucose challenge (mg/dL)	0.08	0.30		−**0.16**	**0.03**

**Figure 2:**
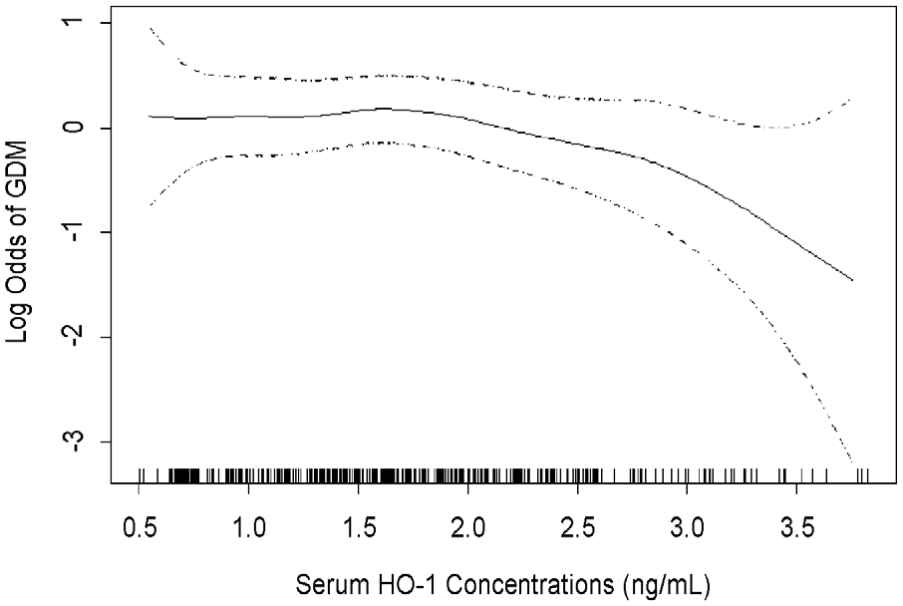
Relation between maternal serum HO-1 concentrations and the adjusted relative odds of gestational diabetes mellitus (GDM) (solid line), with 95% CI (dotted lines). The vertical bars along the serum HO-1 concentrations axis indicate distribution of study subjects. The estimates were adjusted by maternal age, race/ethnicity, family history of diabetes and pre-pregnancy body mass index. (Excluded 3 subjects with serum HO-1 measurements>4 ng/mL, all are non-GDM.).

The unadjusted ORs of GDM decreased across increasing quartiles of maternal serum HO-1 (p-value for linear trend = 0.02) (**Table 3**). After adjustment for maternal age, non-white race, family history of diabetes and pre-pregnancy BMI, early-pregnancy HO-1 concentrations were inversely associated with GDM risk, though the association was no longer statistically significant. Of note, the adjusted OR for the highest vs. lowest quartile for HO-1 concentrations was 0.58 (95% CI 0.30–1.11). We also compared women with the highest concentrations of serum HO-1 concentrations (i.e., those with values in the upper decile of the distribution of H0-1 concentrations among controls) to these women with the lowest HO-1 concentrations (i.e., women in the lowest quartile). After adjusting for confounders, extremely high HO-1 (top decile>3.05 ng/mL) was associated with a 74% decreased odds of GDM (OR = 0.26; 95% CI 0.09–0.77). This finding corresponds to the observation from our flexible dose response model (i.e., from GAM models) and related splines (**Figure 2**), where we noted an inverse association of GDM risk with increasing HO-1 concentrations, particularly when concentrations exceeded 3 ng/mL (**Figure 2**).

**Table 3:** The odds ratio (ORs) and 95% confidence intervals (CI) of gestational diabetes mellitus (GDM) risk according to different levels of maternal serum HO-1 at early pregnancy.

Serum HO-1(ng/mL)	GDM(n = 186)	Controls(n = 191)	UnadjustedOR (95%CI)	Adjusted^1^OR (95%CI)
Quartile 1 (<1.23)	55 (29.6)	47 (24.6)	1.00 (referent)	1.00 (referent)
Quartile 2 (1.23–1.79)	60 (32.2)	48 (25.1)	1.07 (0.62–1.84)	1.15 (0.64–2.07)
Quartile 3 (1.80–2.40)	42 (22.6)	48 (24.1)	0.75 (0.42–1.32)	0.96 (0.51–1.80)
Quartile 4 (≥2.41)	29 (15.6)	48 (25.1)	0.52 (0.28–0.94)	0.58 (0.30–1.11)
*P for trend*			*0.018*	*0.10*
Quartile 1 (<1.23)	55 (29.6)	47 (24.6)	1.00 (referent)	1.00 (referent)
Upper Decile (≥3.05)	6 (3.2)	20 (10.5)	0.26 (0.10–0.69)	0.26 (0.09–0.77)

1 OR and 95% CI adjusted for maternal age, race/ethnicity, family history of diabetes and pre-pregnancy body mass index.

## Discussion

To the best of our knowledge, the current study is the first to examine the relationship between circulating HO-1 concentrations and GDM risk. Furthermore, our study is particularly significant because the relationship was assessed prospectively. We found that an elevated serum HO-1 concentration in early pregnancy is associated with decreased risk of subsequent GDM.

As an inducible stress protein, HO-1 is widely accepted to be a highly sensitive and reliable marker of oxidative stress [12]. Up-regulation of HO-1 protein is thought to represent an attempt to minimize cellular injury. Notably, cells isolated from HO-1 knock-out mice demonstrate lower resistance to oxidative stress [24]. And it was further confirmed in a typical two-allele HO-1 deficiency human case where enhanced endothelial injury was observed following oxidative stress [25]. A recent report also noted a parallel increase in HO-1 protein levels, HO activity, and the levels of serum adiponectin, a protein hormone that is known to modulate a number of metabolic processes, including improved insulin sensitivity and reduced adiposity [26]. The report that HO-1 regulates mitochondrial transport carriers and function [27] suggests that HO-1, by activating Bcl-2 and Bcl-x_L_, prevents cytochrome *c* release and activation of caspases. Collectively, these results suggest that it may be possible to favorably modulate the balance between pro- and anti-apoptotic mechanisms. The HO-1 system has been shown to regulate T-cell proliferation and immune response [28], [29]. A reduction in antioxidant reserves has been related to endothelial cell dysfunction in diabetes [30]. Increased levels of HO-1, through gene transfer in hyperglycemic rats, resulted in a decrease of endothelial cell sloughing [31]. Delivery of the human HO-1 gene to endothelial cells attenuated glucose-mediated oxidative stress, DNA damage, and cell death [32].

Few studies reported that circulating levels of HO-1 were increased in glucose intolerance [17] or T2DM [18] as well as other chronic diseases related to oxidative stress such as silicosis [33], secondary hemophagocytic syndrome (HPS) or adult-onset Still’s disease [34] and Parkinson’s disease [35]. However, Mateo reported that median serum levels of HO-1 did not differ significantly between Alzheimer’s disease patients and controls [35]. Most of the studies were cross-sectional. For example, our results were different from what Bao observed in patients with impaired glucose regulation or T2DM from 2 case control studies. Bao and his colleague [17] found plasma HO-1 concentration was significantly increased among non-diabetic individuals with impaired-glucose-regulation compared with healthy controls (1.34 (0.81–2.29) ng/mL versus 0.98 (0.56–1.55) ng/mL, P<0.001). In an earlier study, Bao reported that plasma HO-1 concentrations were significantly increased in newly diagnosed-T2DM cases compared to controls (median (IQR): 2.42 (1.39–3.90) ng/mL in cases vs. 1.11 (0.63–2.06) ng/mL in controls, *P*<0.001). The ORs for incident T2DM in the highest quartile of plasma HO-1 concentrations, compared with the lowest, was 8.23 (95% CI 5.55–12.21; *P* for trend<0.001) [18]. It needs to be noted, however, that these studies assessed serum HO-1 after or at the time of the clinical diagnosis of T2DM. Consequently, given the cross sectional nature of their study design, it is not possible to clarify the temporal relationship of altered HO-1 synthesis and release with the development of T2DM.

In the current study, we observed that increased serum HO-1 concentrations were associated with low dietary heme iron intake/red meat consumption among women who were euglycemic throughout pregnancy; however, this negative correlation was disrupted in women who subsequently developed GDM. High levels of dietary heme iron intake during the pre-pregnancy, preconceptionally and early pregnancy periods are associated to increased GDM risk [36]. In our study, those who developed GDM had higher heme iron intake/red meat consumption (heme iron: 0.83 vs. 0.76 mg/day; red meat: 0.64 vs. 0.58 servings/day) which is consistent to previous report. And, further adjusting for the above factors in the logistic regression model did not change our estimates materially (data not shown).

Our study has several strengths. First, determination of HO-1 concentrations using serum collected in early pregnancy served to define a relationship between the biomarker and subsequent risk of GDM. Second, the high follow-up rate (>95%) of women enrolled in our study minimized possible selection bias. However, several limitations also merit discussion and consideration. First, a single measurement of serum HO-1 concentrations is not likely to provide a time-integrated measure of maternal circulating HO-1 levels during the entire pregnancy. Thus, longitudinal studies with serial measurements of circulating HO1 concentrations along with indices of insulin sensitivity and secretion across gestation are needed to elucidate the mechanisms and pathophysiological consequences of maternal oxidative stress during pregnancy. Second, as with all observational studies, although we adjusted for known and suspected confounders, we cannot exclude the possibility of residual confounding from unmeasured covariates. Third, universal glucose tolerance testing in early pregnancy is not part of the standard obstetric care. Hence, we cannot exclude the possibility that some subjects in our study had undiagnosed pre-gestational diabetes. However, over 95% of study subjects reported having regular medical exams within a 24-month period before the index pregnancy; and the cumulative incidence of GDM in our study cohort is consistent with observations in other settings [1]. Fourth, the source of the circulating HO-1 still remains uncertain, more careful metabolic studies to help isolate the source(s) of HO-1 are warranted. Finally, the generalizability of our finding is limited to a largely non-Hispanic White, well -educated obstetric population.

In summary, this report extends the current literature by documenting a relation between early pregnancy serum HO-1 concentrations and GDM risk. Large-scale longitudinal studies with multiple point measurements throughout pregnancy, however, are warranted to further exam the extent to which early pregnancy circulating HO-1 concentrations and/or the change of HO-1 concentration during pregnancy might be used as a risk marker for GDM.
